# Association of MicroRNA-149 Polymorphism with Lung Cancer Risk in Chinese Non-Smoking Female: A Case-Control Study

**DOI:** 10.1371/journal.pone.0163626

**Published:** 2016-09-29

**Authors:** Hang Li, Yangwu Ren, Lingzi Xia, Ruoyi Qu, Lingchao Kong, Zhihua Yin, Baosen Zhou

**Affiliations:** 1 Department of Epidemiology, School of Public Health, China Medical University, Shenyang, PR China; 2 Key Laboratory of Cancer Etiology and Intervention, University of Liaoning Province, Shenyang, PR China; Duke Cancer Institute, UNITED STATES

## Abstract

**Introduction:**

Rs2292832 is a single nucleotide polymorphism located in the precursor of mir-149 and was reported to be associated with varieties of malignancies. So far, the effect of miR-149 rs2292832 polymorphism on lung cancer risk was unclear. In addition, cooking oil fume exposure was demonstrated to be an important environmental risk factor in Chinese female. The aim of the present study was to evaluate the associations of rs2292832 polymorphism, cooking oil fume exposure and multiplicative interaction of cooking oil fume exposure and rs2292832 polymorphism with lung cancer risk in Chinese non-smoking female population.

**Methods:**

The present study was a hospital-based case-control study conducted in Chinese non-smoking females. 555 lung cancer patients and 395 cancer-free controls were interviewed to collect demographic data and exposure status of environmental risk factors, and then donate 10 ml venous blood which was used to be genotyped by Taqman allelic discrimination method. The statistical analyses were performed on SPSS 13.0 software.

**Results:**

The association between miR-149 rs2292832 polymorphism and risk of lung cancer(TC vs. TT: OR = 1.006, 95%CI = 0.767–1.321, P = 0.963; CC vs. TT: OR = 0.41, 95%CI = 0.532–1.329, P = 0.458; Dominant model: OR = 0.965, 95%CI = 0.745–1.251, P = 0.788; Recessive model: OR = 0.816, 95%CI = 0.528–1.259, P = 0.357, adjusted for age), non-small cell lung cancer(TC vs. TT: OR = 1.006, 95%CI = 0.767–1.321, P = 0.963; CC vs. TT: OR = 0.841, 95%CI = 0.532–1.329, P = 0.458, adjusted for age), lung adenocarcinoma(TC vs. TT: OR = 0.944, 95%CI = 0.700–1.273, P = 0.707; CC vs. TT: OR = 0.801, 95%CI = 0.485–1.323, P = 0.386, adjusted for age) and squamous cell carcinoma(TC vs. TT: OR = 1.025, 95%CI = 0.641–1.638, P = 0.919; CC vs. TT: OR = 0.792, 95%CI = 0.346–1.813, P = 0.581, adjusted for age) were all not statistically significant. Result of Logistic regression showed that the multiplicative interaction of cooking oil fume exposure and rs2292832 polymorphism was not statistically significant (P = 0.063 for lung cancer and P = 0.064 for lung adenocarcinoma).

**Conclusion:**

MicroRNA-149 rs2292832 polymorphism may not be associated with lung cancer risk in Chinese non-smoking female.

## Introduction

Lung cancer, with the highest morbidity, rank the No.1 killer of all kinds of malignancies that the deaths result from lung cancer annually account for the largest proportion of all cancer-related deaths worldwide.[1] In China, approximately 529,153 patients died of lung cancer in 2011, of which 164,721 were female lung cancer patients. [2] Smoking is an established major environmental risk factor for developing lung cancer and the epidemic of tobacco smoking in China may contribute to the increasing incidence of lung cancer in last two decades. Whereas the prevalence of smoking in Chinese women was as low as 2.4% compared with that relative high as 52.9% among Chinese men[3]. Furthermore, nonsmokers constitute approximately 53% of female lung cancer patients and an increasing trend of the proportion of lung cancer in non-smokers was observed by several researches which suggested that other environmental risk factors or genetic background especially play an indispensable role in the development of lung cancer[4–6].

In last decade, accumulating studies focusing on the genetic predisposition of lung cancer observed that SNPs or mutations in microRNAs were associated with lung cancer risk. MicroRNAs are a subset of evolutionarily conserved short non-protein-coding RNA with length of 21–24 nucleotides. [7]MicroRNAs can completely or partially pairing with message RNAs of protein-coding genes thus leading to the degradation or translation repression of the mRNAs, by these processes microRNAs can regulate the expression level of more than 30% protein coding genes at the post-transcription level. MicroRNAs constitute a large regulatory net in cells in which each microRNA may regulate hundreds of target genes and one gene may be regulated by varieties of microRNAs [8]. It was observed that microRNAs were involved in many biological processes including cell proliferation, differentiation, apoptosis and inflammation, the dysfunction of these processes are associated with the carcinogenesis.[9] There’s a growing body of evidence demonstrated that microRNAs may function as tumor suppressor or oncogene, most of microRNAs coding region are located at cancer-related genomic region and abnormal expression of microRNAs were found in varieties of cancer tissues, so it’s of great value for developing target therapy and biomarker of early diagnosis to investigate the relationship of microRNA with cancer[10, 11].

Mutations such as single nucleotide polymorphism (SNP) located in microRNA sequence may affect the maturation of miRNAs or the affinity of miRNAs binding to the target mRNAs, thus may modify the regulation of miRNAs on transcription process and alter the expression level of target gene which may be cancer-related, then affect the susceptibility to cancer or the prognosis of the cancer patients [12]. Emerging evidence demonstrated that rs2292832 C/T polymorphism in pre-miR-149 is associated with varieties of cancer risk [13–16]. In a study conducted by Kim et al. demonstrated that rs2292832 CC/CT genotype carriers have a lower risk of developing liver cancer compared with TT carriers in Korean population[16]. In a population-based case-control study evaluating the associations between miRNA-SNPs and breast cancer risk in Chinese population found that CC genotype carriers of miR-149 rs2292832 have a 0.46-fold decreased risk for breast cancer than TT genotype carriers.[13] There were only two studies focusing on the effect of rs2292832 polymorphism on lung cancer susceptibility carried out in Asian population [17, 18], but reported negative results. Herein, we conduct the present case-control study to investigate the relationship of rs2292832 polymorphism with lung cancer risk in Chinese non-smoking female.

## Materials and Methods

### Study subjects and data collection

The present hospital-based case-control study was carried out in Shenyang city, located in northwest China. 555 diagnosed histologically lung cancer patients were recruited in the case group from Liaoning Cancer Hospital, The First Affiliated Hospital of China Medical University and The general hospital of Shenyang military and 395 healthy non-smoking female were enrolled in the control group from medical examination centers of the hospitals mentioned above in the same period. The inclusion criteria of the cases were as follows: (1) female lung cancer patients who were histologically diagnosed (2) with no previous cancer, metastasized cancer or previous radiotherapy or chemotherapy (3) non-smoking. Them who smoked less than 100 cigarettes in the lifetime were defined as non-smokers, otherwise he is considered as a smoker. The inclusion criteria of controls were consistent with the (2) (3) of the inclusion criteria of cases. Approval of the investigations was obtained from the Institutional Review Board of China Medical University (Contact information for the Ethics Committee of China Medical University: No.77 Puhe Road, Shenyang North New Area, Shenyang, Liaoning Province, P.R. China, Email: songwj@mail.cmu.edu.cn) and all participants signed the informed consent. Every participant was interviewed to collect the demographic data by questionnaire when they were admitted to the hospitals and donated 10 ml of venous blood.

### SNP Identification and genotyping

We isolated genomic DNA samples from the venous blood of all participants by Phenol-chloroform Method. SNP genotyping was performed on an Applied Biosystems 7500 FAST Real-Time PCR System (Foster City, CA, USA) using Taqman^®^ allelic discrimination (Applied Biosystems, Foster City, CA) with primer probe set. Negative controls were included in each run of the genotyping process, two investigators selected 10% of samples randomly and performed the genotyping of these samples a second time and the results were checked to be concordant by different investigators for the sake of quality control.

### Statistical analysis

T test and χ^2^ test were conducted to compare the difference in demographic variables between the case and control group. Hardy-Weinberg equilibrium (HWE) of control group was tested by performing a goodness-of-fit χ^2^ test. The odds ratios (OR) and their 95% confidence intervals (CI) were calculated by unconditional logistic regression analysis to evaluate the association between rs2292832 polymorphism and lung cancer risk or pathological subtype of lung cancer risk. Difference of cooking oil fume exposure distribution in case and control group was compared by χ^2^ test and odds ratios (OR) and their 95% confidence intervals (CI) of association between cooking oil fume exposure and lung cancer risk was calculated by unconditional logistic regression analysis. Multiplicative interaction of cooking oil fume exposure and rs2292832 polymorphism on risk of lung cancer was tested by logistic regression. The power analysis was performed on “Quanto” software version 1.2.4 (University of Southern California, Los Angeles, CA, USA). Statistical analysis was performed on SPSS 13.0 software (SPSS, Inc. Chicago, IL, USA). All of the tests were two-sided and statistical significance was defined as P<0.05.

## Results

### Baseline characteristics

The demographic characteristics of the studying subjects are shown in Table 1. There were 555 lung cancer patients and 395 controls in the present study. Among the lung cancer patients, there were 370 adenocarcinoma, 96 Squamous cell carcinoma and 89 small cell lung cancer in pathologic type of lung cancer. The mean age for case group and control group were 56.77±11.689 and 56.13±11.642 respectively, result of t-test showed that there is no statistical difference in age between case and control group, which suggested that frequency-matching on age was suitable in our study.(t-test: P = 0.406). The result of goodness-of-fit χ^2^ test showed that genotype distributions of rs2292832 among control group was in agreement with Hardy-Weinberg equilibrium (HWE)(P = 0.858 for rs2292832), which suggested that our control group have an appropriate representativeness for the studying population. Compared with non-exposure individuals, those who had cooking oil fume exposure have a 0.848-fold increased risk for developing lung cancer. (OR = 1.848, 95%CI = 1.271–2.687, P<0.001)

**Table 1:** Baseline characteristics of the study subjects.

characteristics	cases n = 555		controls n = 395		P
	No	%	No	%	
**Age(years)**					
≤50	158	28.5	122	30.9	0.715
51–60	165	29.7	115	29.1	
>60	232	41.8	176	40.0	
**Mean±SD**	56.77±11.689		56.13±11.642		0.406
pathologic type					
Adenocarcinoma	370	66.7			
Squamous cell carcinoma	96	17.3			
Small cell lung cancer	89	16.0			
Oil fume exposure					P<0.001
Yes	100	37.5	65	24.4	
No	167	62.5	201	75.6	

### Genotype distribution and lung cancer risk

Results of genotype distributions in case and control group and the relationship of genotypes of rs2292832 with lung cancer risk are showed in Table 2. There were no statistically significant associations between rs2292832 polymorphism and overall risk of lung cancer. (TC vs. TT: OR = 1.006, 95%CI = 0.767–1.321, P = 0.963; CC vs. TT: OR = 0.841, 95%CI = 0.532–1.329, P = 0.458; Dominant model: OR = 0.965, 95%CI = 0.745–1.251, P = 0.788; Recessive model: OR = 0.816, 95%CI = 0.528–1.259, P = 0.357, adjusted for age) Subsequently, we performed subgroup analysis stratified by histopathology type of lung cancer to investigate whether polymorphisms of miR-149 rs2292832 relate with risk of non-small cell lung cancer, lung adenocarcinoma or squamous cell carcinoma group. The results of the subgroup analysis showed that there were no statistically significant associations in NSCLC group(TC vs. TT: OR = 0.959, 95%CI = 0.723–1.272, P = 0.769; CC vs. TT: OR = 0.796, 95%CI = 0.496–1.279, P = 0.346; Dominant model: OR = 0.927, 95%CI = 0.708–1.214, P = 0.583; Recessive model: OR = 0.813, 95%CI = 0.517–1.279, P = 0.371, adjusted for age), lung adenocarcinoma subgroup (TC vs. TT: OR = 0.944, 95%CI = 0.700–1.273, P = 0.707; CC vs. TT: OR = 0.801, 95%CI = 0.485–1.323, P = 0.386; Dominant model: OR = 0.917, 95%CI = 0.689–1.219, P = 0.549; Recessive model: OR = 0.824, 95%CI = 0.510–1.331, P = 0.429, adjusted for age) or squamous cell carcinoma subgroup(TC vs. TT: OR = 1.025, 95%CI = 0.641–1.638, P = 0.919; CC vs. TT: OR = 0.792, 95%CI = 0.346–1.813, P = 0.581; Dominant model: OR = 0.980, 95%CI = 0.625–1.537, P = 0.931; Recessive model: OR = 0.782, 95%CI = 0.354–1.731, P = 0.545, adjusted for age), data are showed in Tables 2 and 3. We performed a stratified analysis stratified by cooking oil fume exposure, but didn’t get any statistically significant results, data are showed in Table 4. Subsequently, we performed a logistic regression to estimate the multiplicative interaction of cooking oil fume exposure with rs2292832 polymorphism in lung cancer and lung adenocarcinoma subgroup, the results showed that the interaction of rs2292832 polymorphism and cooking oil fume exposure in lung cancer and lung adenocarcinoma were not statistically significant (P values were 0.063 in lung cancer and 0.064 in lung adenocarcinoma, respectively).

**Table 2:** Distributions of rs2292832 genotypes and alleles in two groups and their associations with risk of lung cancer and non-small cell lung cancer.

SNP	No of Controls (%)	Lung Cancer			NSCLC		
miR-149 rs2292832		No (%)	OR^a^ [95%CI]	P value	No (%)	OR^a^ [95%CI]	P value
TT	177(44.8)	254(45.8)	1.00(ref)		218(46.8)	1.00(ref)	
TC	176(44.6)	252(45.4)	1.006(0.767–1.321)	0.963	207(44.4)	0.959(0.723–1.272)	0.769
CC	42(10.6)	49(8.8)	0.841(0.532–1.329)	0.458	41(8.8)	0.796(0.496–1.279)	0.346
TC+CC vs TT			0.965(0.745–1.251)	0.788		0.927(0.708–1.214)	0.583
CC vs TC+TT			0.816(0.528–1.259)	0.357		0.813(0.517–1.279)	0.371
T allele	530	760	1.00(ref)	0.525	643	1.00(ref)	0.131
C allele	260	350	0.939(0.772–1.141)		289	0.858(0.704–1.047)	

**OR**^**a**^, adjusted for age Odds Ratio; **SNP**, Single Nucleotide Polymorphism; **NSCLC**, Non-small Cell Lung Cancer

**Table 3:** Distributions of rs2292832 genotypes and alleles in two groups and their associations with risk of lung adenocarcinoma and squamous cell carcinoma.

SNP	No of Controls (%)	Lung Adenocarcinoma			Squamous cell carcinoma		
miR-149 rs2292832		No (%)	OR^a^ [95%CI]	P value	No (%)	OR^a^ [95%CI]	P value
TT	177(44.8)	174(47)	1.00(ref)		44(45.8)	1.00(ref)	
TC	176(44.6)	163(44.1)	0.944(0.700–1.273)	0.707	44(45.8)	1.025(0.641–1.638)	0.919
CC	42(10.6)	33(8.9)	0.801(0.485–1.323)	0.386	8(8.3)	0.792(0.346–1.813)	0.581
TC+CC vs TT			0.917(0.689–1.219)	0.549		0.980(0.625–1.537)	0.931
CC vs TC+TT			0.824(0.510–1.331)	0.429		0.782(0.354–1.731)	0.545
T allele	530	511	1.00(ref)	0.41	132	1.00(ref)	0.660
C allele	260	229	0.914(0.737–1.133)		60	0.927(0.66–1.301)	

**OR**^**a**^, adjusted for age Odds Ratio; **SNP**, Single Nucleotide Polymorphism

**Table 4:** Associations between rs2292832 and lung cancer risk, stratified by oil fume exposure.

Oil exposure	Genotype	Control(%)	Case(%)	OR^a^(95%CI)	P
Non-exposed	TT	93(46.3)	85(50.9)	1.00(ref)	
	CT	86(42.8)	69(41.3)	0.869(0.562,1.344)	0.528
	CC	22(10.9)	13(7.8)	0.628(0.296,1.330)	0.224
	CT+CC vs TT			0.820(0.542,1.240)	0.346
Exposed	TT	26(40.0)	40(40.0)	1.00(ref)	
	CT	34(52.3)	50(50.0)	0.943(0.487,1.827)	0.862
	CC	5(7.7)	10(10.0)	1.278(0.390,4.186)	0.685
	CT+CC vs TT			0.986(0.520,1.870)	0.966

**OR**^**a**^, adjusted for age Odds Ratio

## Discussion

In the present study, we investigated the associations between miR-149 rs2292832 polymorphism and risk of lung cancer in Chinese non-smoking female population. We selected the newly-diagnosed cases as the studying subjects can effectively avoid the Neyman bias. Because the prevalent patients are more likely to include long-term survivors, the exposure status of studying risk factors of the long-term survivors are more likely to be those which can beneficial to survive with lung cancer, so it’s not approximate to use prevalent patients to study the susceptibility of lung cancer. The cases and controls were selected from three hospitals and three medical examination centers, it can avoid the Berkson bias. So it’s more applicable of using newly-diagnosed cases than prevalent cases in our study.

Given that smoking is an established major environmental risk for developing lung cancer, non-smoking female is an ideal population for investigating the potential effect of genetic background on risk of lung cancer. We investigated the relationship of miR-149 rs2292832 polymorphisms with overall lung cancer, but didn’t get any statistically significant results. Subsequently, in order to elucidate whether miR-149 rs2292832 polymorphisms relate with risk of non-small cell lung cancer, lung adenocarcinoma and squamous cell carcinoma, we carried out a stratified analysis, but didn’t get any statistically significant results either. Tian et.al[17] conducted a study to evaluate the relationship of rs2292832 polymorphisms with overall lung cancer risk in Chinese Han population and found no statistically significant associations. T allele frequency of controls and cases in their studying population is 0.6729 and 0.6606 respectively, which is similar to the T allele frequency of the studying population in our study. In different with our study, some studying subjects who were smoking were included in study conducted by Tian et.al, whereas in our study smoking which may be a potential confounding factor was eliminated. Results of the study conducted in Caucasian population by Vinci S et.al [19] also demonstrated that miR-149 rs2292832 polymorphism were not associated with non-small cell lung cancer risk.

In recent years, to elucidate the exact role of microRNAs play in initiation and progression of cancer, researchers all around world have made great efforts, but the exact mechanism of miRNAs exert its effect on carcinogenesis are still largely unknown. Results of many previous studies on varieties of cancer showed that compared with adjacent normal tissues, expression level of microRNAs in cells from cancer tissue are often in deregulation. MicroRNAs which have abnormal upregulated expression level in cancer cells are considered to play oncogene role and in contrast that microRNAs which are down-regulated in expression level are speculated to act as tumor suppressor. MiR-149 was reported to act as both tumor suppressor and oncogene depending on the tumor type. In a study on colorectal cancer, researchers found that miR-149 was downregulated in tissues of colorectal cancer and inversely correlated with expression level of FOXM1 which was regulated by miR-149 in post-transcriptional level, low miR-149 expression was observed to be significantly associated with lymph node or distant metastasis and advanced TNM. Compared with patients with high miR-149 expression, patients with low miR-149 expression showed poorer prognosis.[20] MiR-149 was also reported to be downregulated and may act as tumor suppressor in gastric cancer [21], renal cell carcinoma [22]. In contrast, miR-149 was demonstrated that expression level is elevated and may act as oncogene in progressive nasopharyngeal carcinoma. [23] Results of a study on melanoma showed that elevated miR-149 was found in metastatic melanoma cell which can targets glycogen synthase kinase-3α, resulting in increased expression of Mcl-1 and enhance the ability of melanoma cell to resist apoptosis, so miR-149 act as oncogene in melanoma.[24] A study on non-small cell lung cancer demonstrated that miR-149 directly targeted FOXM1 which may promote the metastasis of the tumor cell. MiR-149 was downregulated in tumor tissue of non-small cell lung cancer compared with adjacent non-cancer lung tissue, ectopic expression of miR-149 can inhibit the process of epithelial-to-mesenchymal transition (EMT) in non-small cell lung cancer cells. [25] It provides important biologically plausible evidence to that miR-149 is involved in lung cancer.

SNPs in microRNAs coding region may affect the ability of microRNA bind to target mRNA or the maturation process of the microRNA. A study on head and neck squamous cell carcinoma conducted by Tu et.al[26] demonstrated that T allele in precursor form of miR-149 may reduce the efficacy of the maturation process from pre-mir-149 to miR-149 compared with C allele, which result in decrease in expression level of miR-149 and in turn may attenuate the suppression effect of miR-149 on cell motility. The results also showed TT genotype carriers of miR-149 rs2292832 had much more possibility of neck nodal metastasis and had a poorer prognosis compared with CT and CC carriers, which may be associated with the low expression of miR-149 in TT carriers. It was reported that CC genotype carriers of miR-149 rs2292832 have lower miR-149-5p expression level than CT and TT genotype carriers in papillary thyroid cancer patients and CC genotype carriers were associated with increased risk of papillary thyroid cancer compared with TT genotype carriers and TT/TC combined genotype. [27]The exact effect of miR-149 rs2292832 on expression of miR-149 in lung malignancies need to be elucidated in further studies.

It is generally acknowledged that the cause of lung cancer is combination of genetic factors and environmental risk factors. Whereas compared with the low exposed rate of active smoking in Chinese female population[3], Chinese women are more common exposed to environmental risk factors like secondhand smoke and cooking oil fume [28], which may contribute to the risk of developing lung cancer. It was reported that 70% of the non-smokers were exposed to secondhand smoke in a typical week[3] and the rate of non-smoking female exposed to secondhand smoke is relatively high in public places and home in China[29]. But in the present study, we can’t achieve the data of environmental tobacco smoke exposure, so we can’t evaluate its effect on lung cancer risk. It was demonstrated that traditional Chinese cooking style such as deep-frying often emit cooking oil fume which contains mutagens and human carcinogens including benzo(a)pyrene, 1,3-butadiene acrolein, and formaldehyde, exposure to cooking oil fume can significantly increase the risk of developing lung cancer in Chinese non-smoking female[28]. Herein, we conducted logistic regression analysis to evaluate the multiplicative interaction of cooking oil fume exposure and miR-149 rs2292832 polymorphism on lung cancer risk in Chinese non-smoking female but we didn’t achieve any statistically significant results. The result of power calculation was 0.9015 for lung cancer group and 0.8828 for non-small cell lung cancer group.

There are some limitations should be considered in the present study. Firstly, this is a hospital-based study, controls were selected from the three medical examination center which may be not approximate representative for the overall non-smoking population. Secondly, the data of cooking oil fume exposure was collected by the anamnesis of participants which was subjected to recall bias. Thirdly, the sample size may be a barrier for achieving an enough statistical power to get a significant association, especially the samples size of substratum were too small in the subgroup analysis stratified and the result of the power calculation was 0.7802 for non-exposed of cooking oil fume and 0.5609 for exposed of cooking oil fume subgroup in Table 4. So it is still needed to be elucidated in studies with larger sample size in future.

## Conclusions

In conclusion, in the present study, we evaluate association between miR-149 rs2292832 polymorphism and susceptibility of lung cancer in Chinese non-smoking female, our result seems to demonstrate that rs2292832 polymorphism was not associated with lung cancer risk.
